# Safety and Efficacy of Eltrombopag and Romiplostim in Myelodysplastic Syndromes: A Systematic Review and Meta-Analysis

**DOI:** 10.3389/fonc.2020.582686

**Published:** 2020-11-26

**Authors:** Fanqiao Meng, Xiuqiong Chen, Shunjie Yu, Xiaotong Ren, Zhaoyun Liu, Rong Fu, Lijuan Li

**Affiliations:** ^1^Hematology Department of Tianjin, Medical University General Hospital, Tianjin, China; ^2^Cancer Center, Tongji Hospital, Tongji Medical College, Huazhong University of Science and Technology, Wuhan, China

**Keywords:** eltrombopag, meta-analysis, myelodysplastic syndromes, romiplostim, thrombocytopenia

## Abstract

**Background and Aim:**

Many studies indicated that eltrombopag and romiplostim could improve hematopoietic function in patients with myelodysplastic syndromes (MDS), but their toxicity and efficacy were not known. This meta-analysis aimed to investigate the safety and efficacy of eltrombopag and romiplostim in MDS.

**Methods:**

A full-scale search strategy was used to search relevant published studies in PubMed, Embase, Web of Science, ClinicalTrials.gov and the Cochrane Library until January 2020 using a random-effects model and the pooled risk ratio (RR) with 95% confidence interval as the effect indicator. Statistical analyses were performed using RevMan 5.3.

**Results:**

This meta-analysis included eight studies comprising 1047 patients. A lower RR of overall response rate (ORR) (RR: 0.65; 95% CI, 0.47–0.9) and grade ≥3 bleeding events (RR: 0.36; 95% CI, 0.36–0.92) were observed after romiplostim and eltrombopag treatment compared with placebo. The pooled RR for the ORR and grade ≥3 bleeding events were 0.58 (95% CI: 0.41–0.83, P = 0.003) and 0.6 (95% CI: 0.37–0.96, P = 0.03) in eltrombopag, respectively. A lower ORR in intermediate- or high-risk MDS (RR: 0.63; 95% CI: 0.45–0.88, P = 0.006) was observed. No difference in mortality, serious adverse events, platelet transfusion, hematologic improvement, and AML transformation was observed.

**Conclusions:**

Thrombopoietin receptor agonists (TPO-RAs) romiplostim and eltrombopag were effective in reducing bleeding events, especially grade ≥3 bleeding events. However, it might reduce the ORR of MDS, especially in eltrombopag treatment group or high-risk MDS group. Due to the limited treatment of MDS and the poor response to the drug, this may be a selection method for MDS combined with fatal bleeding, although further research is needed to confirm the effectiveness of this approach.

## Introduction

Myelodysplastic syndrome (MDS) is a group of heterogeneous diseases with abnormal quality and quantity of blood cells. It originates from hematopoietic stem cells and is characterized by cytopenia, dysfunctional hematopoiesis, and an increased risk of progression to acute myeloid leukemia (AML) (1–3). Anemia, bleeding, infection, and other symptoms lead to a significant decline in the quality of life of patients, directly resulting in death (4, 5), and treatment should be individualized (6, 7). Thrombocytopenia is a challenge in MDS and is associated with shortened survival and an increased risk of progression to AML (8, 9). Thrombocytopenia is an independent adverse risk factor in MDS (9), is associated with life-threatening bleeding and is common in MDS (10). Therapeutic options for MDS with thrombocytopenia are limited, platelet transfusion is the currently commonly used treatment, but the therapeutic effect is limited, and some patients have serious adverse reactions (11). Patients with MDS having severe thrombocytopenia may benefit from the effective recovery of platelets (12). Therefore, new treatments of thrombocytopenia in MDS remain a medical need. Although some progress has been made in the treatment of MDS, effective treatment for MDS is still lacking (13, 14).

The thrombopoietin receptor agonists (TPO-RAs) romiplostim and eltrombopag selectively interact with thrombopoietin receptors and speed up the proliferation and differentiation of megakaryocytes for treating immune thrombocytopenia (15), AML (16–18), chronic myeloid leukemia (19), and aplastic anemia (20). *In vitro* studies on the effect of eltrombopag on MDS suggested that eltrombopag displayed a beneficial effect on megakaryopoiesis in patients with MDS and without any adverse effect on the survival of bone marrow mononuclear cells (21). Eltrombopag mediates anticancer effects by its ability to chelate iron and modulate intracellular iron homoeostasis (22). TPO-RAs combined with azacytidine, lenalidomide, or decitabine could alleviate hematologic toxicity and improve platelet counts. However, some studies reported that it was detrimental to patients with MDS. In a phase 3 study on patients with MDS, eltrombopag was not conducive to platelet recovery, with lower response rates and a trend toward increased progression to AML (23).

The safety and efficacy of TPO-RAs in MDS are still inconclusive due to the dissimilarity in results and hence need to be confirmed. Therefore, this systematic meta-analysis was performed to evaluate the safety and efficacy of eltrombopag and romiplostim in patients with MDS.

## Methods

### Literature Search

This meta-analysis was conducted in accordance with the Cochrane Handbook for Systematic Reviews, Preferred Reporting Items for Systematic Review and Meta-Analysis (PRISMA) statement (24) and was registered with PROSPERO (CRD42020215619). PubMed, Embase, Web of Science, ClinicalTrials.gov and the Cochrane Library were systematically searched from inception to January 2020, without language restriction. The medical subject heading terms were as follows: ((((("Myelodysplastic Syndromes"[Mesh]) OR (Dysmyelopoietic Syndromes)) OR (Hematopoetic Myelodysplasia)) OR (Syndromes, Dysmyelopoietic)) OR (Myelodysplasias, Hematopoetic)) AND (((((“Amgen Megakaryopoiesis protein 531”) OR (Nplate)) OR (AMG531)) OR (romiplostim)) OR ((((Promacta) OR (SB-497115)) OR (Revolade)) OR (eltrombopag))).

### Study Selection and Data Abstraction

Full study analysis and data extraction were reviewed independently by two investigators FM and XC. The inclusion criteria were as follows: (1) randomized controlled trials (RCTs) with more than 10 patients in one arm and (2) RCTs on the treatment of MDS with eltrombopag or romiplostim. Studies including individual case reports, letters, single-arm studies, case-control studies, reviews, studies reporting other diseases than MDS, clinical trials with no results, and nonhuman researches were excluded. The following characteristics were extracted: the first author’s name, publication time, condition, age, sample size, clinical trial ID, sex (male), study sponsor, outcome measures and treatments.

### Outcome Measures

The primary endpoint was overall response rate (ORR) according to the International Working Group criteria of complete or partial response (25). The secondary endpoints included bleeding, serious adverse events (SAE), serious treatment-related adverse events, adverse events ≥3, death, platelet transfusion (PT), hematologic improvement (HI), platelet hematologic improvement (HI-P), erythroid hematologic improvement (HI-E), neutrophil hematologic improvement (HI-N), and AML transformation.

### Statistical Analysis

According to the Cochrane Handbook for Systematic Reviews of Interventions, the following criteria were used to assess the risk of bias: sequence generation, allocation concealment, blinding (participants, personnel, and outcome assessors), incomplete outcome data, selective outcome reporting, and other sources of bias. All statistical analyses were conducted using RevMan version 5.3. A *P* value less than 0.05 was considered statistically significant. The heterogeneity was assessed using *I*^2^ values: low (*I*^2^ = 0%–25%), medium (*I*^2^ = 25%–50%), high (*I*^2^ = 50%–75%), and nonignorable (*I*^2^ = 75%–100%). There will be a clinical heterogeneity between studies included in this study. A random-effects model was used to calculate the pooled results. The subgroup and sensitivity analyses were conducted to analyze the heterogeneity among studies.

## Results

### Search Results

**Figure 1:**
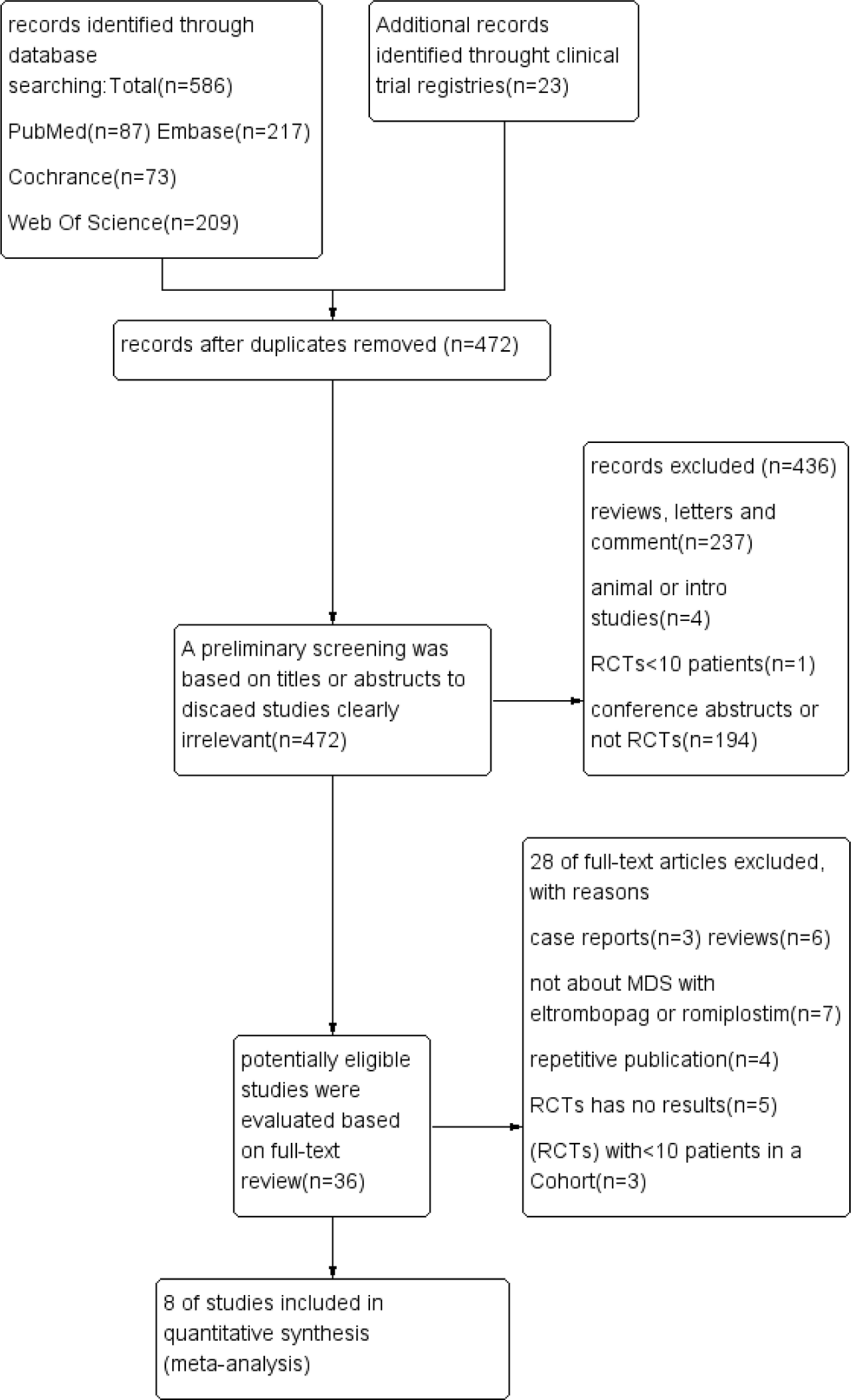
Flow diagram showing the search and data extraction.

As illustrated in **Figure 1** and **Table 1**, 609 unique studies were identified during the initial search: PubMed (n = 87), Embase (n = 217), Cochrane Library (n = 73), Clinical trial registries (n=23) and Web of Science (n = 209). After removing 137 duplicate studies, 472 remained for further screening. A preliminary screening was based on titles or abstracts to discard studies clearly irrelevant. Then 36 potentially eligible studies were evaluated based on full-text review. As a result, 28 studies were excluded, and the remaining eight were included in the meta-analysis.

**Table 1:** Searching strategy.

Databases	Number of article found	Number of article included	Number of excluded article	Reason for exclusion
PubMed	N = 87	N = 8	N = 79	Reviews, letters and comment(N = 36), not RCTs (N = 42),RCTs<10 patients (N = 1)
Web of Science	N = 209	N = 8	N = 201	case reports (N = 3),not RCTs (N = 73), duplicate studies (N = 62), Reviews, letters and comment (N = 63)
Cochran Library	N = 73	N = 7	N = 66	Reviews, letters and comment (N = 55), not about MDS with TPO-RAs (N = 3), duplicate studies (N = 8)
Embase	N = 217	N = 8	N = 209	Reviews, letters and comment (N = 83), not RCTs (N = 79), duplicate studies (N = 43), not human studies (N = 4)
Embase	N = 23	N = 8	N = 15	not about MDS with TPO-RAs (N = 7), RCTs have no result (N = 5), RCTs<10 patients (N = 3)

### Characteristics of Included Studies

All eight studies were RCTs: four on the use of eltrombopag compared with placebo (17, 23, 26, 27) and another four on the use of romiplostim (28–31). This meta-analysis involved 1047 participants [657 (63%) male]; most of them were White/Caucasian and adults. The studies were published between 2010 and 2018, and the sample size ranged from 29 to 356. All patients were diagnosed with MDS on the basis of the World Health Organization (WHO) criteria (32). Three studies included patients with a low risk MDS (only intermediate-1) (26, 29, 31), three studies included patients with middle risk MDS (intermediate-1 and intermediate-2) (23, 28, 30),and two trials included patients with high risk MDS(high risk MDS and AML patients) (17, 27). The percentage of platelet count <50 in four studies was more than 50% (23, 28, 30, 31). The characteristics of the eight included studies are described in **Tables 2** and **3**.

**Table 2:** Study characteristics.

Study	Year	Clinical trial ID	Number	Median age	Male (%)	IPSS<=1(%)	disease	Caucasian	Funding
Kantarjian et al. (31)	2018	NCT00614523	250	70	148 (59%)	250 (100%)	MDS	235(94%)	Amgen Inc
Greenberg et al. (28)	2013	NCT00321711	29	68	19 (66%)	14 (48%)	MDS	20 (69%)	Amgen Inc
Kantarjian et al. (30)	2010	NCT00321711	40	71	24 (60%)	26 (65%)	MDS	37 (93%)	Amgen Inc
Dickinson (23)	2018	NCT02158936	356	70	234 (66%)	125 (35%)	MDS	294 (83%)	Novartis Pharma AG
Oliva et al. (26)	2017	EudraCT201002289033	90	69	52 (58%)	90 (100%)	MDS	NA	Associazione QOL-ONE
Wang et al. (29)	2012	NCT00418665	38	74	24 (62%)	35 (90%)	MDS	36 (92%)	Amgen Inc
Mittelman (17)	2018	NCT01440374	145	72	97 (67%)	0 (0)	MDS+AML	126 (87%)	Novartis Pharma AG
Platzbecker et al. (27)	2015	NCT00903422	98	NA	59 (60%)	NA	MDS+AML	68 (70%)	GlaxoSmithKline

NA, not available; MDS, myelodysplastic syndromes; AML, acute myeloid leukemia; IPSS, international prognostic scoring system.

**Table 3:** The treatments and outcomes of the included studies.

Study	Treatments	Trial interventions	Outcomes
Dickinson (23)	Eltrombopag plus azacitidine	Eltrombopag (start, 200 mg/d [East Asians,100 mg/d], maximum, 300 mg/d [East Asians, 150 mg/d]) or placebo, plus azacitidine (75 mg/m^2^ subcutaneously once daily for 7 days every 28 days)	The primary end point was the proportion of patients who were free of PT during cycles 1 through 4 of azacytidine therapy. Secondary end points included OS, disease response, duration of response, progression to AML and PFS, HI, safety, and tolerability.
Oliva et al. (26)	Eltrombopag	Eltrombopag (50 mg to 300 mg) or placebo for at least 24 weeks and until disease progression and were masked to treatment allocation.	The primary endpoints were the proportion of patients achieving a PR within 24 weeks and safety. Secondary endpoints included time to response, PT, incidence and severity of bleeding, changes in quality-of-life score.
Mittelman (17)	Eltrombopag	Eltrombopag or placebo at 100 mg per day (50 mg per day for patients of east-Asian heritage) to a maximum of 300 mg per day (150 mg per day for patients of east-Asian heritage).	disease response; HI; PFS; maximum PT independence duration from weeks 5 to 12; WHO Bleeding Scale-based bleeding; DP; OS; and quality of life assessment; PT
Platzbecker et al. (27)	Eltrombopag	Once daily eltrombopag or matching placebo dose adjusted from 50 mg to a maximum dose of 300 mg.	The primary endpoint includes AE, nonhematological laboratory grade 3–4 toxic effects, and changes in bone-marrow blast counts from baseline. Secondary end points were PR, PT, OS, and plasma eltrombopag concentration.
Wang et al. (29)	Romiplostim plus lenalidomide	Weekly placebo or romiplostim 500 μg or 750 μg for four 28-day lenalidomide cycles.	AE, bleeding events, and concomitant medications, progression to AML, CSTEs, and PT, percentage of patients who had a reduction or delay in lenalidomide, CR, PR, or OR and incidence of bleeding events
Greenberg et al. (28)	Romiplostim plus decitabine	Romiplostim 750 μg or placebo and decitabine.	The primary end point was CSTEs. Secondary objectives were to evaluate the safety and tolerability of romiplostim in combination with a hypomethylating agent; the proportion of patients receiving hypomethylating agent treatment and schedule; PT
Kantarjian et al. (30)	Romiplostim plus azacitidine	Romiplostim 500 g or 750 g or placebo subcutaneously once weekly during 4 cycles of azacitidine.	The primary endpoint was CSTEs. Secondary endpoints incidence of PT frequency and number of units transfused, incidence of azacitidine dose reduction, or delay resulting from thrombocytopenia, and response rate at the end of azacitidine treatment.
Kantarjian et al. (31)	Romiplostim	Placebo or 750 μg romiplostim subcutaneously once per week for 58 weeks.	The primary outcomes were survival and progression to AML. CSTEs, PT, bleeding events, and PR, OS, AE

CSTEs, incidence of clinically significant thrombocytopenic events; DP, disease progression; HI, hematologic improvement; PT, platelet translation; OS, overall survival; PFS, progression-free survival; CR, complete response, PR, partial response.

### Quality Assessment

**Figure 2:**
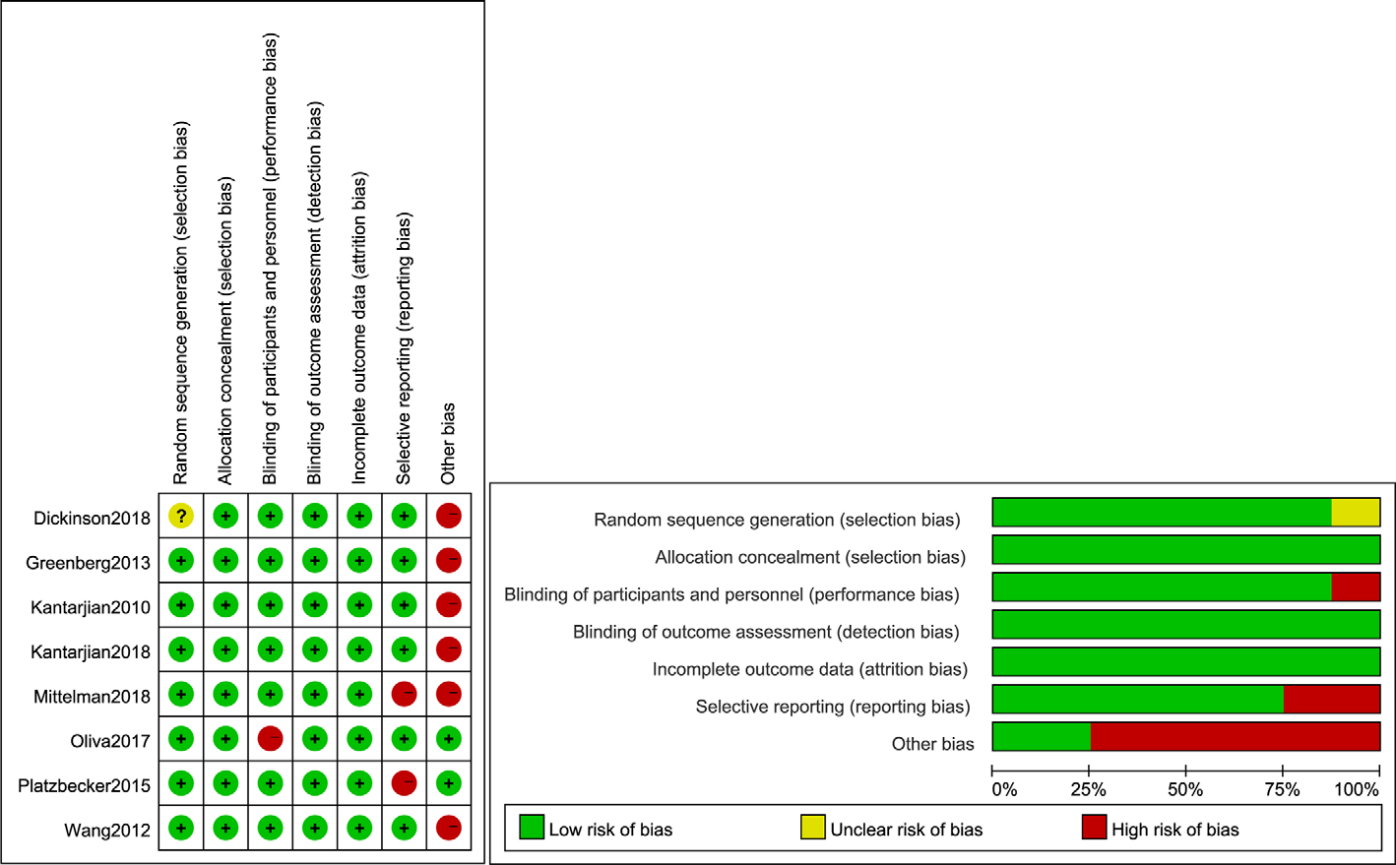
Quality assessment of the included comparative studies. +, Low risk of bias; –, high risk of bias; ?, unclear risk of bias.

The quality assessment details for the studies are graphically summarized in **Figure 2**. The high risk originated from other biases as inevitable limitations and defects in the study. Although some aspects of the assessment studies were risky, the overall risk of bias was not high.

### Overall Response Rate

**Figure 3:**
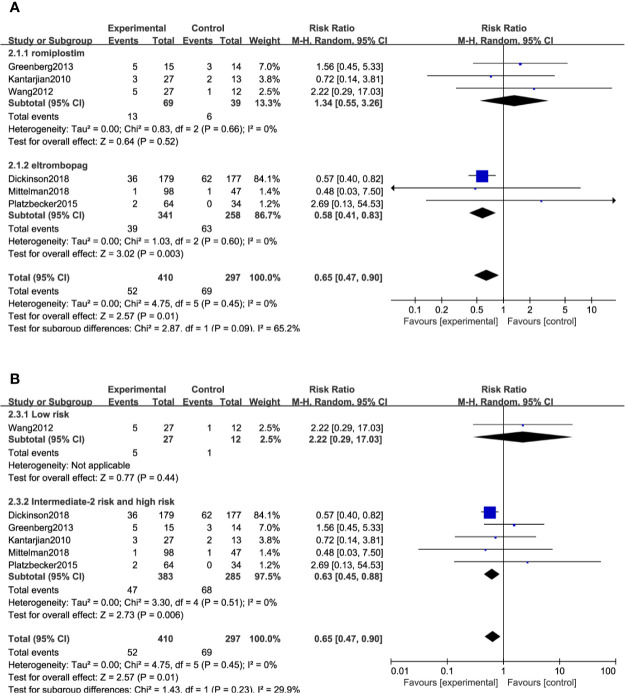
Subgroup analysis of the ORR based on TPO-Ras **(A)** and MDS risk groups **(B)**.

All included studies reported the ORR except two trials (26, 31). A total of 707 (TPO-RAs/placebo: 410/297) patients were enrolled. TPO-RAs significantly reduced the ORR compared with placebo, with a pooled RR rate of 0.65 (95% CI: 0.47–0.9, *P* = 0.01) using the random-effects model (**Figure 3**). Despite no significant heterogeneity (*I*^2^ = 0%, *P* = 0.45), a subgroup analysis was performed based on different types of TPO-RAs and MDS risk groups. Of note, the pooled RR for the ORR was 0.58 (95% CI: 0.41–0.83, *P* = 0.003) in the case of eltrombopag, but for romiplostim, the pooled RR for the ORR was 1.34 (95% CI: 0.55–3.26, *P* = 0.52). The subgroup analysis revealed a significant difference for ORR in intermediate- or high-risk MDS (RR: 0.63; 95% CI: 0.45–0.88, *P* = 0.006), but no significant difference in low risk MDS (RR: 2.22; 95% CI: 0.29–17.03, *P* = 0.44), compared to placebo (**Figure 3B** and **Table 4**). Results of sensitivity analysis showed no study resulting in the heterogeneity, indicating that TPO-RAs significantly reduced the ORR, especially in eltrombopag treatment group or high-risk MDS group.

**Table 4:** The subgroup analysis in different types of MDS risk groups.

	Low risk MDS			Intermediate or high-risk MDS		
	RR (95% CI)	P	Heterogeneity	RR (95% CI)	P	Heterogeneity
**ORR**	2.22 (0.29–17.03)	0.44	NA	0.63 (0.45–0.88)	0.006	I^2^ =0, P=0.51
**Bleeding** **events**	0.66 (0.27–1.61)	0.36	I^2^=67%, P=0.05	0.92 (0.81–1.03)	0.15	I^2^ =0, P=0.67
**Grade >= 3** **bleeding** **events**	NA	NA	NA	0.58 (0.36–0.92)	0.02	I^2^ =0, P=0.84
**AML** **progression**	1.18 (0.59–2.38)	0.64	I^2^ =0, P=0.58	1.07 (0.72–1.58)	0.73	I^2^=28%, P=0.24
**Serious** **adverse** **events**	0.89 (0.28–2.78)	0.84	I^2^=87%, P=0.005	0.97 (0.72–1.31)	0.84	I^2^=72%, P=0.01
**Mortality**	1.03 (0.81–1.30)	0.82	I^2^ =0, P=0.98	0.97 (0.70–1.36)	0.88	I^2^=36%, P=0.18

ORR, overall response rate; NA, not available.

### Bleeding Events

**Figure 4:**
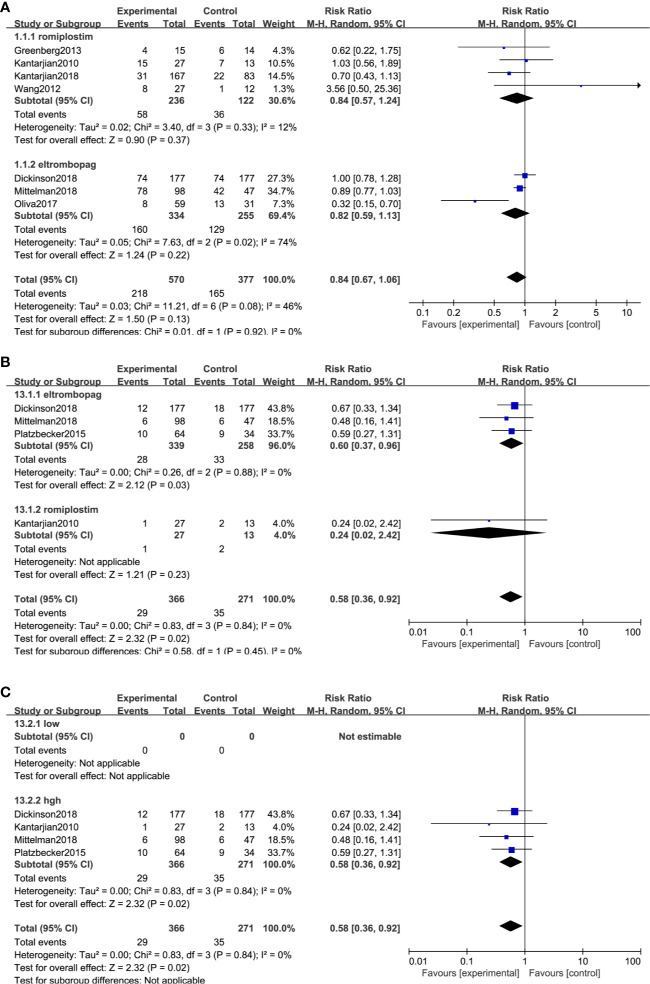
TPO-RAs subgroup analysis of the number of patients with bleeding events **(A)**. Subgroup analysis of the grade ≥3 bleeding events based on TPO-Ras **(B)** and MDS risk groups **(C)**.

Bleeding events were compared in two ways: the number of patients who happened bleeding events and grade >= 3 bleeding events. Seven trials, including 947 patients, reported bleeding events, and 4 trials reported grade ≥3 bleeding events. The result indicated medium heterogeneity among bleeding events (*I*^2^ = 46%, *P* = 0.08) and no significant heterogeneity among grade ≥3 bleeding events (*I*^2^ = 0%, *P* = 0.84). TPO-RAs reduced the risk of bleeding events compared with placebo (RR: 0.84; 95% CI: 0.67–1.06, *P* = 0.13) but with no significant difference (**Figure 4A**). However, for grade ≥3 bleeding events, the results showed a significant difference (RR: 0.58; 95% CI: 0.36–0.92, *P* = 0.02), indicating that TPO-RAs significantly reduced grade ≥3 bleeding events (**Figure 4B**). The subgroup analysis revealed a significant difference for grade ≥3 bleeding events in the case of eltrombopag (RR: 0.6; 95% CI: 0.37–0.96, P = 0.03), but no significant difference in the case of romiplostim (RR: 0.24; 95% CI: 0.02–2.42, P = 0.23), compared to placebo. Significant difference was found in grade ≥3 bleeding events in high-risk MDS groups (RR: 0.58; 95% CI: 0.36–0.92, P = 0.02), compared to placebo (**Figure 4C** and **Table 4**).

### AML Progression

**Figure 5:**
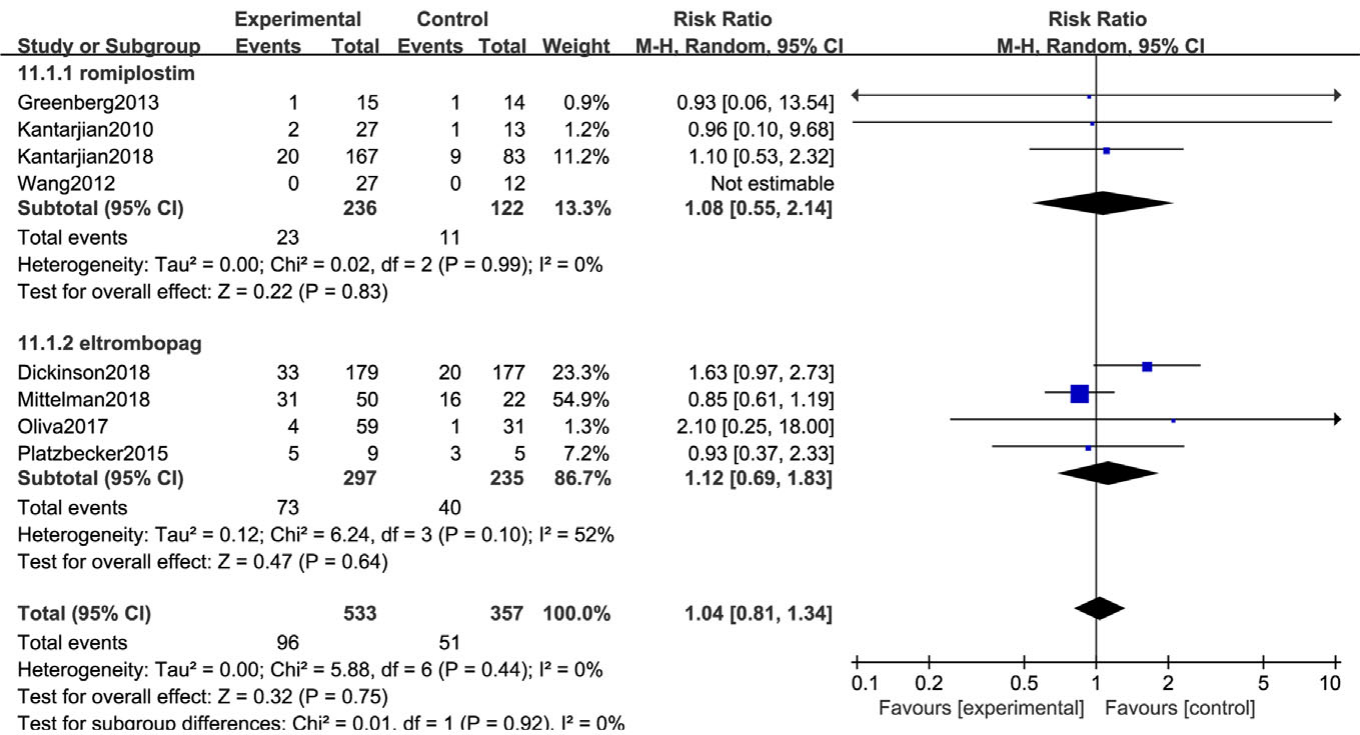
TPO-RAs subgroup analysis of AML progression.

All 8 trials with a total of 890 patients reported the risk of AML7nbsp;progression. No significant difference in transformation into AML (RR: 1.04; 95% CI: 0.81–1.34, *P* = 0.75) was observed in placebo versus TPO-RAs (**Figure 5**). Despite low heterogeneity (*I*^2^ = 0%, *P* = 0.44), the subgroup analyses in MDS risk groups revealed no significant differences (**Table 4**).

### Other Outcomes

**Figure 6:**
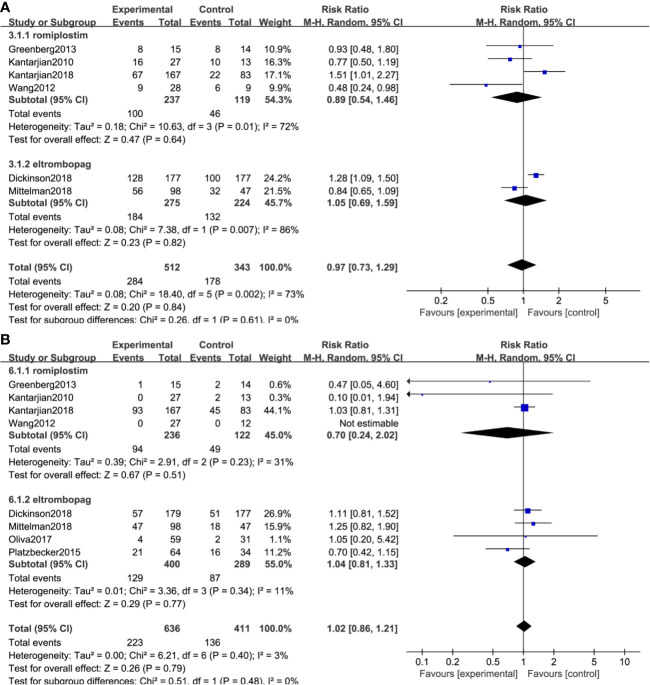
TPO-RAs subgroup analysis of serious adverse events **(A)** and death events **(B)**.

No significant difference in serious adverse events (RR: 0.97; 95% CI: 0.73–1.29, P = 0.84) and mortality (RR: 1.02; 95% CI: 0.86–1.21, *P* = 0.79) was observed in placebo versus TPO-RAs (**Figures 6A, B**). No statistically significant difference was found in platelet transfusion, serious adverse events, serious treatment-related adverse events, and adverse events ≥3 between placebo and TPO-RAs. All the other outcomes are described in **Table 5**.

**Table 5:** Statistical analysis of other outcomes.

Outcome	Subgroup RR (95% CI)Total		RR (95% CI)	Total	
	Eltrombopag	Romiplostim		P	Heterogeneity
CSTEs	NA	0.85 (0.67–1.07)	0.85 (0.67–1.07)	0.17	0
Mortality	1.04 (0.81-1.33)	0.70 (0.24–2.02)	1.02 (0.86–1.21)	0.79	3%
DP	0.97 (0.65-1.36)	NA	0.97 (0.65–1.36)	0.86	46%
HI	1.05 (0.81-1.38)	NA	1.05 (0.81–1.38)	0.7	0
HI-E	NA	1.27 (0.59–2.70)	1.27 (0.59–2.70)	0.54	51%
HI-P	1.89 (0.43-8.28)	3.42 (0.23–50.27)	2.40 (0.77–7.44)	0.13	85%
HI-N	1.04 (0.24-4.50)	3.02 (0.79–11.47)	1.48 (0.50–4.33)	0.48	52%
SAE	1.05 (0.69-1.59)	0.89 (0.54–1.46)	0.97 (0.73–1.29)	0.84	73%
STRAE	1.10 (0.55-2.21)	0.47 (0.10–2.18)	0.95 (0.50–1.79)	0.88	0
AE>=3	1.11 (0.80-1.53)	0.92 (0.65–1.29)	1.04 (0.83–1.31)	0.71	45%
PT	1.00 (0.65-1.53)	0.70 (0.47–1.06)	0.88 (0.63–1.23)	0.45	74%

CSTEs, incidence of clinically significant thrombocytopenic events; DP, disease progression; HI, hematologic improvement; HI-E, erythroid hematologic improvement; HE-P, platelet hematologic improvement; HI-N, hematologic improvement neutrophil; SAE, serious adverse events; AE>=3, adverse events>=3; PT, platelet translation; STRAE, serious treatment-related adverse events; NA, not available.

### Publication Bias and Sensitivity Analysis

**Figure 7:**
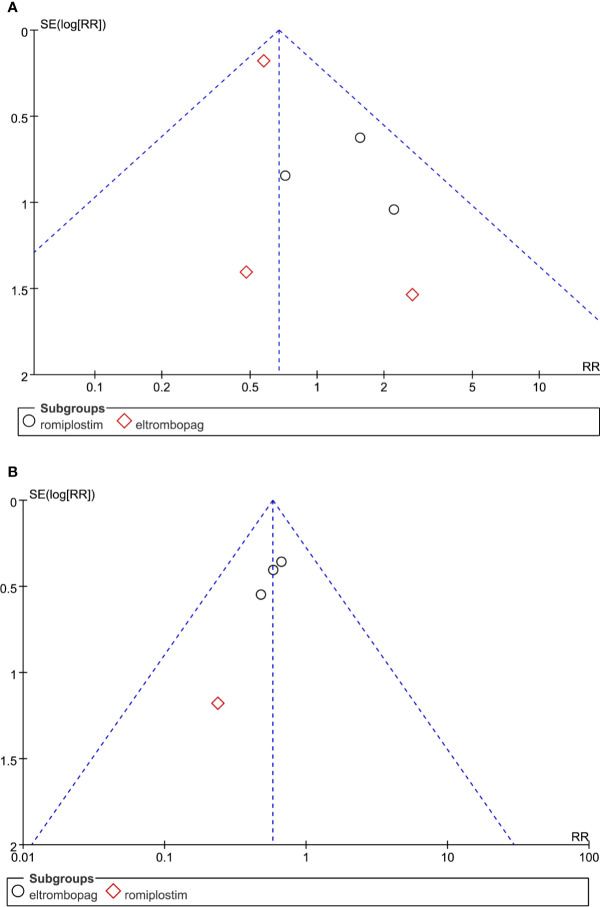
Funnel plot analysis of potential publication bias in the study: **(A)** ORR and **(B)** grade ≥3 bleeding events.

From above results, we can see ORR and grade ≥3 bleeding events were significant statistically. So we performed sensitivity analysis and publication bias for these two indicators. Sensitivity tests found no significant impact on the stability of meta-analysis at the ORR and grade ≥3 bleeding events when one study was omitted. Funnel plot analysis of publication bias suggested that there was potential publication bias in ORR (**Figure 7A**), because funnel plots showed slight non-symmetry. No significant publication bias was observed in grade ≥3 bleeding events (**Figure 7B**).

## Discussion

The use of TPO-RAs for treating MDS is still under investigation, with some results encouraging and some disappointing (33). Hence, the ORR of TPO-RAs, the reduction of bleeding and AML transformation, and the effectiveness and safety of TPO-RAs in the treatment of MDS remain controversial. Therefore, this analysis was performed to explore the efficacy of TPO-RAs in the treatment of MDS and provide clinical references. Our meta-analysis indicated that TPO-RAs significantly reduced the ORR (RR = 0.65; 95% CI: 0.47–0.9, *P* = 0.01). However, Vicente et al report that eltrombopag monotherapy can improve hematopoiesis in patients with low to intermediate risk-1 MDS. Eleven of 25 (44%) patients responded; five and six patients had hematologic responses, respectively (34). The possible explanations were that patients with refractory anemia with excess blasts, AML, treatment-related MDS, or chronic myelomonocytic leukemia were excluded, while patients in our meta-analysis were thrombocytopenia with advanced MDS or AML. In addition, each trial had different treatment backgrounds and final points. Two trials were discontinued due to the potential risk for progression to AML. Only the intermediate- or high-risk MDS group reported ORR in detail, which may affect the results. Importantly, TPO-RAs significantly reduce ORR in intermediate- or high-risk MDS (RR: 0.63; 95% CI: 0.45–0.88, P = 0.006).Funnel plot find significant publication bias in ORR. Based on these results, we do not recommend that TPO-RAs be routinely used in MDS therapy, especially in the high-risk MDS group. However, whether TPO-RAs can reduce ORR of MDS needs further exploration.

In our study, in patients treated with eltrombopag (RR: 0.6; 95% CI: [0.37, 0.96], p=0.03), grade >= 3 bleeding events was lower in romiplostim (RR: 0.24; 95% CI: [0.02, 2.42], p=0.23). But it is no direct comparison between eltrombopag and romiplostim, all eight studies are conducted with placebo or others drugs. In addition, four trials reported this outcome, but only one reported romiplostim, with very wide confidence intervals and indirect comparison, thus we cannot consider grade >= 3 bleeding events was lower in romiplostim. But our results indicated that TPO-RAs can remarkably reduce grade >= 3 bleeding events, especially eltrombopag. Our results indicate that TPO-RAs reduced the risk of bleeding events but with no significant difference. TPO-RAs can remarkably reduce grade >= 3 bleeding events rather than bleeding events, the reasons for these discrepancies are evident. The bleeding events reported included minor bleeding, which were not accurately reported, may influence the expected statistical outcome. Furthermore, bleeding events were defined as number of patients with bleeding, not adjusted for exposure per patient month, so there was non-significant. These results were consistent with previous meta-analysis that when adjusted, there was significantly RR of bleeding (35).

In addition, our meta-analysis found no significant differences in mortality and progression to AML. Although TPO-RAs were beneficial to patients with MDS in terms of other outcomes, the difference was not statistically significant (**Table 5**). In addition, significant heterogeneity, wide confidence intervals, very small number of events and two trials were terminated prematurely, which require further investigation. Despite high heterogeneity, sensitivity analysis and publication bias were not performed in these outcomes because no statistically significant difference was found in placebo and TPO-RAs. As we found statistically significant difference in ORR and grade ≥3 bleeding events, we only performed sensitivity analysis and publication bias in these two indicators. No statistical significance was found in sensitivity analysis. When it comes to publication bias, no statistical significances were found in grade ≥3 bleeding events, but potential publication bias was found in ORR. By analyzing the causes of publication bias, it is found that the publication bias is mainly due to the small number of literature inclusion and the small sample size of a few studies. In addition, this meta-analysis included less than 10 trials, so the significance of publication bias is limited.

A meta-analysis involving 746 patients found no significant differences in mortality (RR: 0.97; 95% CI: 0.73–1.27) and progression to AML (RR: 1.02; 95% CI: 0.59–1.77, *P* = 0.03), but a lower risk of bleeding events (RR: 0.92; 95% CI: 0.86–0.99) in MDS treated with TPO-RAs (35), which were also consistent with the results of the present meta-analysis.

The risk of bias for the included studies are assessed in selection bias, performance bias, detection bias, attrition bias, reporting bias and others bias. As shown in **Figure 2**, the high risk of bias originated from other biases and selective reporting. Potential limitations of including studies and different treatment regimens contributed to a high risk of other biases. All trials reported the risk of other biases, except two trials (26, 27). Greenberg et al (2013) used decitabine and Kantarjian et al (2010), Dickinson et al (2018) used azacitidine, both at standard dosing regimens. MDS patients receiving lenalidomide in study by Wang et al. Patients were randomly assigned to receive eltrombopag (50–300 mg) (26, 27), (100–300 mg) (17), (200–300 mg) (23), and romiplostim (750 µg) (28, 31), (500 and 750 µg) (29, 30). Two trials (17, 27) included both patients with MDS and patients with AML, and hence were judged with a high risk for selective reporting. In addition, only one trial was single-blind (26), which also contributed to a high risk of bias. However, the results were not significantly different when sensitivity analysis was performed. Therefore, the overall risk of bias was not high.

This meta-analysis had several limitations. First, data on some outcomes were insufficient. Further, two studies included patients with AML, leading to a potential risk of bias. Second, although a comprehensive search strategy was used, relevant studies were unavoidably missed, especially those published in a language other than English. Third, the random-effects model used in this meta-analysis might have minimized the inherent variances.

In conclusion, TPO-RAs were effective in reducing bleeding events, especially grade ≥3 bleeding events. However, it might reduce ORR of the MDS, especially in eltrombopag treatment group or high-risk MDS group. More studies with larger sample sizes and long-term follow-up are needed to evaluate the safety and efficacy of TPO-RAs in MDS. Although further studies are needed, our meta-analysis suggests that TPO-RAs is not recommended for high risk MDS patients unless combined with fatal bleeding.

## Data Availability Statement

The original contributions presented in the study are included in the article/supplementary material, further inquiries can be directed to the corresponding author.

## Author Contributions

FM and XC designed the study, performed the analysis, and wrote the paper. SY, ZL, and XR abstracted the data and assisted in the collection and analysis of the data. LL and RF critically revised the manuscript and ensured a correct analysis of the data. All authors contributed to the article and approved the submitted version.

## Funding

This study was partly sponsored by the National Natural Science Fund of China (Grant Number: 81770118).

## Conflict of Interest

The authors declare that the research was conducted in the absence of any commercial or financial relationships that could be construed as a potential conflict of interest.

## References

[1] Garcia-Manero G Fenaux P Hypomethylating agents and other novel strategies in myelodysplastic syndromes. J Clin Oncol (2011) 29(5):516–23. 10.1200/JCO.2010.31.0854 PMC3056493 21220589

[2] Garcia-Manero G Myelodysplastic syndromes: 2015 Update on diagnosis, risk-stratification and management. Am J Hematol (2015) 90(9):831–41. 10.1002/ajh.24102 26294090

[3] Jabbour E Takahashi K Wang X Cornelison AM Abruzzo L Kadia T Acquisition of cytogenetic abnormalities in patients with IPSS defined lower-risk myelodysplastic syndrome is associated with poor prognosis and transformation to acute myelogenous leukemia. Am J Hematol (2013) 88(10):831–7. 10.1002/ajh.23513 PMC3923606 23760779

[4] Kim SY Park Y Kim H Kim J Kwon GC Koo SH Discriminating myelodysplastic syndrome and other myeloid malignancies from non-clonal disorders by multiparametric analysis of automated cell data. Clin Chim Acta (2018) 480:56–64. 10.1016/j.cca.2018.01.029 29378171

[5] Kattamis A Aydinok Y Taher A Optimising management of deferasirox therapy for patients with transfusion-dependent thalassaemia and lower-risk myelodysplastic syndromes. Eur J Haematol (2018) 101(3):272–82. 10.1111/ejh.13111 29904950

[6] Zeidan AM Faltas B Douglas Smith B Gore S Myelodysplastic syndromes: what do hospitalists need to know? J Hosp Med (2013) 8(6):351–7. 10.1002/jhm.2049 PMC4234094 23666619

[7] Sekeres MA Epidemiology, natural history, and practice patterns of patients with myelodysplastic syndromes in 2010. J Natl Compr Canc Netw (2011) 9(1):57–63. 10.6004/jnccn.2011.0006 21233244

[8] Kantarjian H Giles F List A Lyons R Sekeres MA Pierce S The incidence and impact of thrombocytopenia in myelodysplastic syndromes. Cancer (2007) 109(9):1705–14. 10.1002/cncr.22602 17366593

[9] Gonzalez-Porras JR Cordoba I Such E Nomdedeu B Vallespi T Carbonell F Prognostic impact of severe thrombocytopenia in low-risk myelodysplastic syndrome. Cancer (2011) 117(24):5529–37. 10.1002/cncr.26173 21638279

[10] Basood M Oster HS Mittelman M Thrombocytopenia in Patients with Myelodysplastic Syndromes: Still an Unsolved Problem. Mediterr J Hematol Infect Dis (2018) 10(1):e2018046. 10.4084/MJHID.2018.046 30002802 PMC6039085

[11] Nishiuchi T Okutani Y Fujita T Yoshida K Ohnishi H Haba R Effect of iron chelator deferasirox on chronic anemia and thrombocytopenia in a transfusion-dependent patient with myelodysplastic syndrome. Int J Hematol (2010) 91(2):333–5. 10.1007/s12185-010-0500-5 20127527

[12] Tang Y Zhang X Han S Chu T Qi J Wang H Prognostic Significance of Platelet Recovery in Myelodysplastic Syndromes With Severe Thrombocytopenia. Clin Appl Thromb Hemost (2018) 24(9_suppl):217S–22S. 10.1177/1076029618802363 PMC6714828 30296835

[13] Patel B Hirsch C Clemente M Sekeres M Makishima H Maciejewski JP Genetic and molecular characterization of myelodysplastic syndromes and related myeloid neoplasms. Int J Hematol (2015) 101(3):213–8. 10.1007/s12185-015-1747-7 25690487

[14] Stahl M Zeidan AM Hypomethylating agents in combination with histone deacetylase inhibitors in higher risk myelodysplastic syndromes: Is there a light at the end of the tunnel? Cancer (2017) 123(6):911–4. 10.1002/cncr.30532 28094843

[15] Kim TO Despotovic J Lambert MP Eltrombopag for use in children with immune thrombocytopenia. Blood Adv (2018) 2(4):454–61. 10.1182/bloodadvances.2017010660 PMC5858473 29487060

[16] Shastri A Verma AK Eltrombopag reduces clinically relevant thrombocytopenic events in higher risk MDS and AML. Lancet Haematol (2018) 5(1):e6–7. 10.1016/S2352-3026(17)30229-6 29241763

[17] Mittelman M Platzbecker U Afanasyev B Grosicki S Wong RSM Anagnostopoulos A Eltrombopag for advanced myelodysplastic syndromes or acute myeloid leukaemia and severe thrombocytopenia (ASPIRE): a randomised, placebo-controlled, phase 2 trial. Lancet Haematol (2018) 5(1):34–43. 10.1016/s2352-3026(17)30228-4 29241762

[18] Gangatharan SA Cooney JP Persistent thrombocytopenia post auto-SCT for AML treated with romiplostim in a patient with HIV. Bone Marrow Transplant (2011) 46(9):1280–1. 10.1038/bmt.2010.298 21151181

[19] Ramadan H Duong VH Al Ali N Padron E Zhang L Lancet JE Eltrombopag Use in Patients With Chronic Myelomonocytic Leukemia (CMML): A Cautionary Tale. Clin Lymphoma Myeloma Leuk (2016) 16:S64–S6. 10.1016/j.clml.2016.02.009 27521328

[20] Gill H Leung GMK Lopes D Kwong Y-L The thrombopoietin mimetics eltrombopag and romiplostim in the treatment of refractory aplastic anaemia. Br J Haematol (2017) 176(6):991–4. 10.1111/bjh.14024 27097929

[21] Mavroudi I Pyrovolaki K Pavlaki K Kozana A Psyllaki M Kalpadakis C Effect of the nonpeptide thrombopoietin receptor agonist eltrombopag on megakaryopoiesis of patients with lower risk myelodysplastic syndrome. Leuk Res (2011) 35(3):323–8. 10.1016/j.leukres.2010.06.029 20688394

[22] Roth M Will B Simkin G Narayanagari S Barreyro L Bartholdy B Eltrombopag inhibits the proliferation of leukemia cells *via* reduction of intracellular iron and induction of differentiation. Blood (2012) 120(2):386–94. 10.1182/blood-2011-12-399667 PMC3398759 22627766

[23] Dickinson M Cherif H Fenaux P Mittelman M Verma A Portella MSO Azacitidine with or without eltrombopag for first-line treatment of intermediate- or high-risk MDS with thrombocytopenia. Blood (2018) 132(25):2629–38. 10.1182/blood-2018-06-855221 PMC6337824 30305280

[24] Liberati A Altman DG Tetzlaff J Mulrow C Gotzsche PC Ioannidis JP The PRISMA statement for reporting systematic reviews and meta-analyses of studies that evaluate healthcare interventions: explanation and elaboration. BMJ (2009) 339:b2700. 10.1136/bmj.b2700 19622552 PMC2714672

[25] Tefferi A Cervantes F Mesa R Passamonti F Verstovsek S Vannucchi AM Revised response criteria for myelofibrosis: International Working Group-Myeloproliferative Neoplasms Research and Treatment (IWG-MRT) and European LeukemiaNet (ELN) consensus report. Blood (2013) 122(8):1395–8. 10.1182/blood-2013-03-488098 PMC4828070 23838352

[26] Oliva EN Alati C Santini V Poloni A Molteni A Niscola P Eltrombopag versus placebo for low-risk myelodysplastic syndromes with thrombocytopenia (EQoL-MDS): phase 1 results of a single-blind, randomised, controlled, phase 2 superiority trial. Lancet Haematol (2017) 4(3):e127–e36. 10.1016/S2352-3026(17)30012-1 28162984

[27] Platzbecker U Wong RSM Verma A Abboud C Araujo S Chiou TJ Safety and tolerability of Eltrombopag versus placebo for the treatment of thrombocytopenia in patients with advanced myelodysplastic syndromes or acute myeloid leukaemia: a multicentre, randomised, placebo-controlled, double-blind, phase 1/2 trial. Lancet Haematol (2015) 2(10):e417–26. 10.1016/S2352-3026(15)00149-0 26686043

[28] Greenberg PL Garcia-Manero G Moore M Damon L Roboz G Hu K A randomized controlled trial of romiplostim in patients with low-or intermediate-risk myelodysplastic syndrome receiving decitabine. Leuk Lymphoma (2013) 54(2):321–8. 10.3109/10428194.2012.713477 22906162

[29] Wang ES Lyons RM Larson RA Gandhi S Liu DL Matei C A randomized, double-blind, placebo-controlled phase 2 study evaluating the efficacy and safety of romiplostim treatment of patients with low or intermediate-1 risk myelodysplastic syndrome receiving lenalidomide. J Hematol Oncol (2012) 5:13. 10.1186/1756-8722-5-71 23190430 PMC3520696

[30] Kantarjian HM Giles FJ Greenberg PL Paquette RL Wang ES Gabrilove JL Phase 2 study of romiplostim in patients with low- or intermediate-risk myelodysplastic syndrome receiving azacitidine therapy. Blood (2010) 116(17):3163–70. 10.1182/blood-2010-03-274753 PMC3324162 20631375

[31] Kantarjian HM Fenaux P Sekeres MA Szer J Platzbecker U Kuendgen A Long-term follow-up for up to 5 years on the risk of leukaemic progression in thrombocytopenic patients with lower-risk myelodysplastic syndromes treated with romiplostim or placebo in a randomised double-blind trial. Lancet Haematol (2018) 5(3):e117–e26. 10.1016/S2352-3026(18)30016-4 29396092

[32] Vardiman JW Harris NL Brunning RD The World Health Organization (WHO) classification of the myeloid neoplasms. Blood (2002) 100(7):2292–302. 10.1182/blood-2002-04-1199 12239137

[33] Svensson T Chowdhury O Garelius H Lorenz F Saft L Jacobsen SE A pilot phase I dose finding safety study of the thrombopoietin-receptor agonist, eltrombopag, in patients with myelodysplastic syndrome treated with azacitidine. Eur J Haematol (2014) 93(5):439–45. 10.1111/ejh.12383 24853277

[34] Vicente A Patel BA Gutierrez-Rodrigues F Groarke E Giudice V Lotter J Eltrombopag monotherapy can improve hematopoiesis in patients with low to intermediate risk-1 myelodysplastic syndrome. Haematologica (2020). 10.3324/haematol.2020.249995 PMC7716353 33256377

[35] Dodillet H Kreuzer KA Monsef I Skoetz N Thrombopoietin mimetics for patients with myelodysplastic syndromes. Cochrane Database Syst Rev (2017) 9:CD009883. 10.1002/14651858.CD009883.pub2 28962071 PMC6483680

